# Development of Microplatforms to Mimic the In Vivo Architecture of CNS and PNS Physiology and Their Diseases

**DOI:** 10.3390/genes9060285

**Published:** 2018-06-06

**Authors:** John Saliba, Arij Daou, Samar Damiati, Jessica Saliba, Marwan El-Sabban, Rami Mhanna

**Affiliations:** 1 Biomedical Engineering Program, American University of Beirut (AUB), Beirut 1107 2020, Lebanon; jes11@mail.aub.edu (J.S.); ad75@aub.edu.lb (A.D.); 2 Department of Biochemistry, Faculty of Science, King Abdulaziz University (KAU), Jeddah 21589, Saudi Arabia; sdamiati@kau.edu.sa; 3 Department of Biology, Faculty of Science, Lebanese University, Beirut 6573/14, Lebanon; jessicasaliba@hotmail.com; 4 Department of Anatomy, Cell Biology and Physiological Sciences, Faculty of Medicine, American University of Beirut (AUB), Beirut 1107 2020, Lebanon; me00@aub.edu.lb

**Keywords:** organ-on-a-chip, nervous tissues, cell co-cultures, blood-brain barrier, neurodegenerative diseases, brain cancer, metastasis

## Abstract

Understanding the mechanisms that govern nervous tissues function remains a challenge. In vitro two-dimensional (2D) cell culture systems provide a simplistic platform to evaluate systematic investigations but often result in unreliable responses that cannot be translated to pathophysiological settings. Recently, microplatforms have emerged to provide a better approximation of the in vivo scenario with better control over the microenvironment, stimuli and structure. Advances in biomaterials enable the construction of three-dimensional (3D) scaffolds, which combined with microfabrication, allow enhanced biomimicry through precise control of the architecture, cell positioning, fluid flows and electrochemical stimuli. This manuscript reviews, compares and contrasts advances in nervous tissues-on-a-chip models and their applications in neural physiology and disease. Microplatforms used for neuro-glia interactions, neuromuscular junctions (NMJs), blood-brain barrier (BBB) and studies on brain cancer, metastasis and neurodegenerative diseases are addressed. Finally, we highlight challenges that can be addressed with interdisciplinary efforts to achieve a higher degree of biomimicry. Nervous tissue microplatforms provide a powerful tool that is destined to provide a better understanding of neural health and disease.

## 1. Introduction

### 1.1. Nervous System Cells: Their Roles and Microenvironment

The design of biomimetic platforms to study neural physiology requires an understanding of the native structure of these tissues including cells, matrix and their interactions. The functioning brain tissue is constituted from several types of cellular elements that are anatomically integrated. Of particular significance to this review are two classes of cells, neurons and glia, which are interconnected via very complex circuitries [1]. Neurons and glia exist in the brain in approximately equal numbers; however, neurons are responsible for most of the brain’s unique functions. The neuron itself is the fundamental element and the backbone of the nervous tissue. Glial cells on the other hand, also called the “sleeping giants” of neuroscience, have highly diverse and incompletely understood roles [1,2,3]. We know however that without glia, the brain cannot function properly as they play a huge role in maintaining, processing and supporting neuronal functionalities. Several classes of neuroglia exist; these include astrocytes (the most numerous), oligodendroglia, microglia and Schwann cells. Their roles include myelination (Schwann and oligodendroglia), enveloping synaptic junctions to control neurotransmitter overspread in the synaptic cleft (astrocytes), regulating concentrations of certain ions in the extracellular medium (astrocytes), scavenging molecular and cellular debris in addition to dead or degenerating neurons and glia (microglia), secretion of trophic factors (astrocytes), as well as development and maintenance of the (BBB) [3,4]. In addition to neuroglia and neurons, ependymal cells exist in ventricular regions to direct cell migration during brain development.

All our thoughts, behaviors, emotions and actions are encoded at the level of complex neuron-neuron interactions as well as the circuits where these interactions take place and that constitute the structural basis for brain function. Neurons can be categorized according to their morphology, size, topographic location, neurochemistry, and more importantly connectivity [5]. Information delivered to the peripheral nervous system (PNS) is carried by motor neurons, and information delivered back to the brain is carried by primary sensory neurons, whereas interneurons (the most numerous in the nervous tissues) form connections only with other neurons [6,7]. All of these factors are important determinants of the role the neurons play within the tissues they reside in. Population of neurons projecting from one region to another form macrocircuits, whereas local neuron-neuron interactions within a brain region form microcircuits [7,8,9]. Neurons are embedded with membrane-spanning proteins known as ion channels that allow the passage of specific charged particles (ions) through their pores and that are gated via complex mechanisms. It is the passage of these ions that generates voltage, the currency of the brain. Ion channels are the maestros of neuronal functionalities and the secret to decode the brain lies at their behalf. If we need to understand the neuronal basis of healthy and diseased brains, we need to understand how ion channels cooperate to generate voltage as well as these macro- and microcircuits interactions. Thus, these cellular and circuit characteristics allow us to appreciate the special structural and biochemical qualities a neuron exhibits in relation to its surroundings [1].

Morphologically, three major regions can be defined in a typical neuron [10]: (1) the cell body (soma or cyton), which contains the nucleus and other important cytoplasmic organelles (like the rough endoplasmic reticulum, the smooth endoplasmic reticulum, the Golgi apparatus, and the mitochondria); (2) dendrites, which emanate from the soma as antennas and are covered with thousands of synapses (the wide variety of dendritic shapes and branches are used to classify different groups of neurons); and (3) axon, an extrusion from the soma which tapers away much farther than dendritic branches do [1,10]. Axons are generally smooth, emit one or more collaterals, and are surrounded by the myelin sheath that facilitates rapid impulse conduction. The end of the neuron, called the axon terminal, is the site where the neuron comes in contact with other neurons (or other cells) and passes signals to them. This point of contact is called the synapse [1,10]. The synapse has two sides: presynaptic and postsynaptic (the names indicate the usual direction of information flow from “pre” to “post”). The presynaptic side is basically the axon terminal, whereas the postsynaptic side can be a dendrite or the soma of another neuron. These factors display a wide range of morphological specializations, depending on the target area in the central or peripheral nervous system. Therefore, in mimicking a physiologic or pathophysiologic condition one needs to take into account the actual composition of cells and their surrounding matrix for the target area.

### 1.2. Microfluidic and Organ-on-Chip Technology

**Figure 1:**
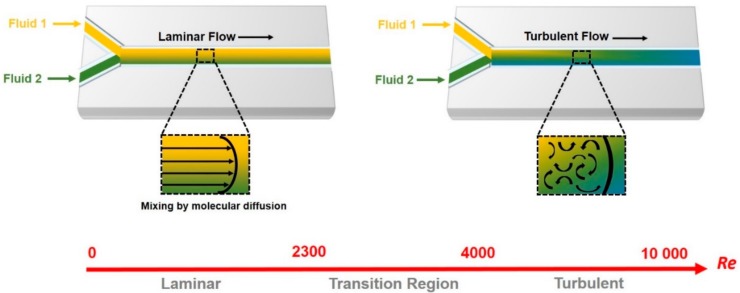
Schematic showing the laminar and turbulent flow. The Reynolds number (Re) describes the physical characteristics of the fluid flow in microfluidic channels. In laminar flow (Re < 2300), the two streams move in parallel to the flow direction and mixed based on the diffusion (**Left**). In turbulent flow (Re > 4000), fluids move in all three-dimensions without correlation with the flow direction (**Right**). The transition region (2300 < Re < 4000) shares the features of laminar and turbulent flow.

The field of microfluidics (key to the development of lab-on-a-chip technology) first appeared in the 1980s, and since that time has attracted increasing attention as a promising field merging the areas of science and device engineering [11,12,13]. Microfluidic platforms, as an alternative to two-dimensional (2D) cell cultures, enable cell growth, reactions, and analysis on the same device, in turn reducing cost, time, and labor [14,15]. Microfluidic systems may consist of parallel, splitting, and merging channels besides functional units such as pumps, mixers, reactors, valves, and porous membranes [16,17]. Moreover, microfluidic chips can be integrated with biochemical or electrical sensors [18]. The fabrication of micro-miniaturized devices involving multiple channels and chambers enables the investigation of multiple samples in parallel under controlled conditions [19]. The dimensions of the microchannels are usually in the range of tens to hundreds of micrometers, allowing sample/reagent volumes in the range of nano- to pico-liters to be handled. Indeed, small microfluidic tools provide many advantages such as minimal waste production, speedy reaction times (seconds or milliseconds), rapid mass and heat transfer rates, and rapid diffusion [13,20,21]. Enhanced sensitivity, resolution, and precision in microfluidic systems may be attributed to high surface-to-volume ratio in the microstructure [22]. Several surface-related properties guiding the microfluidic system include laminar flow, surface tension, electrokinetics, and thermal response time [23]. When fluids are confined in microchannels their physics differ, and hence, the relationship between the inertial and viscous forces can be calculated by the Reynolds number (Re) according to the following formula:

Re = ρVDhμ

where *ρ* is the flow density, V is the flow rate, D_h_ is the hydraulic diameter, and µ is the viscosity. Typically, the Re is less than 2300 due to the small dimensions of the microfluidic channels and the fact that the laminar flow is more dominant than the turbulent flow (Figure 1) [24,25,26].

**Figure 2:**
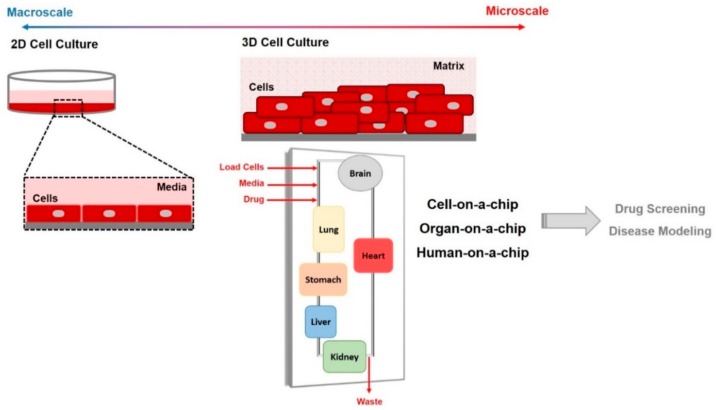
A schematic diagram of traditional two-dimensional (2D) monolayer cell culture and three-dimensional (3D) microfluidic cell culture systems.

Microfluidic technology allows the in vivo organ microenvironment to be mimicked by fabricating a three-dimensional (3D) cell culture that models physiological conditions (Figure 2). The integration of 3D cell culture and cell-based analysis techniques allows for multiple steps such as culture, capture, lysis, and detection of living cells to be performed on the same platform [14,27]. Indeed, 3D cell cultures more closely resemble the in vivo environment with respect to morphology, proliferation, differentiation, and migration. Thus, organ-on-a-chip technology has been exploited to mimic living tissues through the fabrication of the minimal functional units of an organ (Table 1). Developed chips enable the culture of living cells with a continuous supply of oxygen and nutrients as well as a minimal number of components in a microfluidic chamber that is adequate for maintaining interactions at the level of tissues and organs [28]. Hence, organ-on-a-chip platforms allow the investigation of cell behavior by simulating the complex cell–cell and cell–matrix interactions [29]. Depending on the microfluidic architecture and tissue perfusion, biological and physiological reactions can be monitored for approximately one month on the fabricated device [30]. Organ-on-a-chip technology offers many possibilities for investigating cell responses to biochemical and mechanical stimuli from the surrounding environment. Many organ-on-a-chip tools have been fabricated mimicking brain [31], cardiac [32], lung [33], liver [34], kidney [28], and intestinal [35] tissues, and have been used in drug screening assays to evaluate cell response as well as drug efficacy and toxicity [36]. The possibility of connecting organ-on-a-chip platforms with a circulatory system allows for the estimation of drug absorption, distribution, metabolism, and excretion in an in vivo-like model [23]. The engineering of lung tissues into microfluidic channels allows for research into inhaled drug delivery. The toxicity of pharmaceutical compounds can be examined using heart-, gut-, and kidney-on-a-chip devices, while the liver-on-a-chip can be used to examine their toxicity [37]. For the evaluation of drug effects using organ-on-a-chip devices, it is necessary to fabricate special platforms that take into consideration the relevant biological barriers. Multilayered membrane-based microfluidic chips that model biological barriers such as the skin, nasal and small intestine mucosa, as well as the BBB, have been successfully developed [38].

Organ-on-a-chip microenvironments not only aid in improving our understanding of the basic mechanisms governing the function of organs, but also provide a high-throughput human organ simulation model that enables drug screening under specific biomimetic controlled conditions. These 3D platforms aim to replace animals used in clinical research, reduce costs and labor, offer alternative tools with greater flexibility and enable real-time monitoring of dynamic processes. Organ-on-a-chip models of nervous tissues are of paramount importance given the delicate nature of the tissues which limits in vivo experimentation especially in the central nervous system (CNS). The next section reviews models that mimic the CNS and PNS microenvironment to answer basic and applied questions about key neural physiological events.

## 2. Nervous System-on-a Chip Models That Mimic the CNS and PNS Microenvironment and Its Physiology

### 2.1. Neurons and Skeletal Muscle Cells Co-Culture and Neuromuscular Junctions

The neuromuscular junction (NMJ) is a synapse of the PNS which has long been used as a model for the basic principles of synapse development and maintenance due to its experimental accessibility [43]. Functional in vitro NMJs have been developed by co-culturing motoneurons and skeletal muscle fibers [44,45]. These models have contributed to understanding the formation and maturation of the NMJ which will provide insight into clinical solutions for spinal cord injury and neuromuscular diseases [46]. However, these co-cultured models cannot generate functional NMJ since the skeletal muscle cells cannot contract as they are adhered to the culture dishes. These models do not mimic the in vivo niche, especially the 3D architecture of native tissues. NMJ-on-chip devices are being developed to identify the essential interactions and pathways in the formation and maturation of the NMJ and to screen potential drugs [45]. To mimic the in vivo niche of the NMJ, a model was developed to study the pharmacokinetics of NMJ and contractility of muscle fibers [47]. The model was achieved by utilizing tissue engineering methods in synthesizing free-standing muscle fibers. Mouse myoblasts, C2C12 were 3D cultured in patterned Matrigel on polydimethylsiloxane (PDMS) substrates and attached to glass sections. The myoblasts were then differentiated into muscle fibers which were aligned within the Matrigel. Mouse embryonic stem cells (mESC) composed of neurospheres were immobilized onto the muscle fibers by minimizing the culture medium and then differentiating the neurospheres into mature neurons. This model was able to show the difference in the displacement of muscle fibers with electrodes in the presence or absence of active neurons. The contractile displacement of standalone muscle fibers stimulated by electrodes was around 1 µm while that of activated neurons was around 38 µm. This model is, however, limited in its ability to stimulate each cell type independently. In this essence, compartmentalized devices have been of interest to investigate synaptogenesis of NMJ and to dissect the pathways between motoneurons and muscle fibers [48]. A microfluidic device with two compartments separated by microchannels was developed with a compartment for neurons derived from mESC and a compartment for muscle fibers differentiated from C2C12 cells [49]. The microchannels separating the two compartments were 10 µm wide, with a length of 500 µm and height of 2.5 µm. The differentiated motoneurons were able to develop axons that crossed the microchannels into the muscle fiber compartment and formed NMJs. The maintenance of the NMJ in this model through anterograde and retrograde transport was investigated by studying the role of glial-derived neurotrophic factor (GDNF) when added to the different compartments [50]. GDNF showed a positive effect on axonal growth and muscle innervation when added to the muscle fiber compartment while no effect was observed when added to the motoneuron compartment [50]. This model using motoneurons and muscle fibers is a simple representation of the in vivo niche. A similar model was designed to recapitulate the in vivo niche by adding Schwann cells to neurons, along with muscle cells [51]. The model used primary spinal motoneurons and glial cells, and skeletal myocytes taken from Sprague-Dawley rats. The model showed that NMJs were formed from mature motoneurons and that the viability of the motoneurons improved with the addition of glial cells.

### 2.2. Neuron Cultures, Neurogenesis and Synaptic Formation, and Neural Networks

The ability to generate cultures of neuronal cells has been a fundamental challenge but also a fundamental necessity to help advance our understanding of nervous system functionality. Culturing neuronal cells in vitro is challenging because mature neurons do not undergo cell division. Recent advancements in cell culturing have enabled us to study neuronal differentiation, growth factor–dependent cell survival, axon outgrowth, and other basic mechanisms of sensory nerve conduction via dissociated primary sensory neurons [52,53,54,55]. Sensory neurons for culturing can be obtained easily from dorsal root ganglia (DRGs) located along the spinal column, or from cranial sensory ganglia (like trigeminal ganglia (TGs) in cranial nerve V) from mouse or rat embryos shortly after sensory neurons are generated [56,57,58,59,60]. Cultured TGs and DRGs neurons exhibit and share many of the neurophysiological features of the sensory neurons in vivo. They have been used extensively in a wide range of studies including investigation of programmed neuronal death [61,62,63,64], growth factor signaling pathways [62,65,66,67,68], exploring location-specific signaling pathways [69,70], regeneration [71,72], and the role of axonal proteins in modulating synaptic function [73,74,75]. Cultured neurons are used extensively to study neural electrophysiology and they retain the ability to respond to chemical [76,77,78], thermal [79,80] and mechanical [81] stimuli in culture. Microfluidic technology has been used successfully for in vitro sensory neuron culturing to understand how perturbations in the neuron’s microenvironment can affect various neural physiological events including axonal mitochondrial movement among others [82,83,84,85,86]. Moreover, microfluidics are able to isolate the somas of neurons from axons, enabling spatially restricted studies of injury and exposure to changes in pH, neurotoxins and cell-cell communication [87,88,89].

The field of neurogenesis, generation of new neurons, has come a long way in the past two decades [90,91,92,93,94]. For many years however, neuroscientists believed that neurogenesis was restricted to early brain development. It was not until the 1990s that this view was challenged, and scientists started to believe that neural stem cells (NSCs) are incorporated into the adult brain as well [95,96,97]. This mechanism was identified in songbirds [98] but then afterwards discovered in reptiles and fish [99]. In mammals, neurogenesis appears to be a peculiarity of the dentate gyrus (DG) of the hippocampus as well as the olfactory bulb (OB), although new neurons have been reported in other areas (neocortex [100,101,102] and hypothalamus [103]). The ability of NSCs to proliferate and differentiate into all various kinds of cells of the brain necessitates the thorough understanding of these cells and the underlying mechanisms that govern their differentiation. NSCs generate neural progenitor cells (NPCs) which eventually differentiate to neurons or glia. NPCs from the subventricular zone (SVZ) migrate and supply newborn neurons for the OB; those in the subgranular zone (SGZ) migrate a short distance into the DG and integrate into the existing circuitry of the hippocampus. Glial cells play a key role in the development and maintenance of neurogenesis. In particular, interactions between microglia and NPCs regulate neurogenesis via phagocytosis and secretion of cytokines and chemokines [104,105,106,107]. Astrocytes in turn induce neuronal differentiation of NPCs via the release of neurogenic factors [108,109,110]. For example, it has been shown that astrocytes produce brain-derived neurotrophic factor (BDNF) neurotrophins that regulate hippocampal neurogenesis [111,112,113,114]. Neurogenesis is regulated via several mechanisms and at different levels, including the network and local circuit level [115,116], neuromodulatory level like serotonin (5-HT), norepinephrine (NE), dopamine (DA), and acetylcholine (ACh) [117,118,119,120], local signaling level like astrocytes [110], and other extrinsic factors level like exercise, stress and diet [102,109,121,122,123,124]. Recently, 3D neurovascular tissues were constructed by combining in vitro neurogenesis and angiogenesis models using a microfluidic platform [125,126], where a triculture of human NSCs, human brain microvascular ECs (BMECs) and human mesenchymal stem cells (MSCs) was combined [125]. The success of these culture models is very important as it enables not only the investigation of the various neuronal functionalities but also drug screening studies [125,127,128,129,130].

It remains an active area of research to unveil where, when and how synapses are formed (synaptogenesis), as this plays a key role in our understanding of the organization of neuroarchitecture and how circuits are wired and organized to allow information storage and eventually behavior. The formation of neuronal circuitry requires the delicate orchestration of several developmental events including cell fate specification, cell migration, axon guidance, dendritic growth, synaptic target selection, and synaptogenesis [131,132,133,134]. Circuit formation begins with cell fate specification which is regulated via various transcriptional factors and precursor cells. These factors and cells act as conveying points that guide various developmental stages from cell identity to neurite guidance and eventually to synapse assembly [134,135,136]. The extension of polarized projections to become axons and dendrites is the next step after neural cell fate is specified and its accompanying precursors migrate to the corresponding regions. Axons, however, need to be guided to reach their postsynaptic sites. This guidance process is orchestrated by the growth cone which is a “sensing” site existing at the tip of the outgrowing axon [137,138] and is directed by various molecular cues such as Netrin, which regulates neural polarization which then leads to growth cone formation [134,139,140,141]. The “stop” signal that ultimately slows down the growth cone extension to the target cell is calcium/cAMP, which also mediates synaptic differentiation [133,134]. The genesis of the synapse officially starts with the contact and communication between the pre- and the postsynaptic partners which is mediated by cell surface adhesion molecules like NCAM, laminin and cadherins [131,142,143,144,145,146], and which are all Ca^++^ dependent.

### 2.3. Neuron and Glial Co-Culture and Neuron-Glial Interactions

Conventional Transwell-based systems commonly used to study neuron-glia interactions are far from actually mimicking the in vivo conditions, especially when specific axon-glia interactions are of interest. Signaling exchange between neurons and glial cells is not limited to the synapse but can be mediated by nitric oxide released from axons which stimulate myelin basic protein phosphorylation in oligodendrocytes [147].

In an attempt to mimic such interactions, Park and co-workers designed a multi-compartment microfluidic platform to spatially segregate neuronal cell bodies from axons and glial cells [148]. Briefly, somatic bodies were confined to a larger compartment (the soma compartment), which connects to the axon-glia compartment by arrays of axon-guiding microchannels. Six independent axon-glia compartments are built within the same microfluidic unit, allowing for the conduct of six independent experiments simultaneously. This novel system was tested using primary neurons isolated from Sprague-Dawley rats which were loaded onto the soma compartment. One week later, axons had crossed the microchannels into the axon-glia compartment. By the end of the second week, oligodendrocytes were added to the axon-glia compartment for co-culture. The authors concluded the successful fabrication of a microfluidic system that allows for localized axon-glia interaction studies. The system was then employed by the same group to investigate axon myelination in the CNS. Two weeks after seeding neurons obtained from embryonic rats, axons had filled over half of the axon microchannels. Oligodendrocyte progenitors from neonatal rats were added to the axon-glia compartments; they differentiated into mature oligodendrocytes, suggesting that this microfluidic system serves studies investigating axon-glia interaction and signaling [149]. They further characterized the use of this platform by adding astrocytes to the axon-glia compartment. Unlike oligodendrocyte progenitors which thrived and differentiated, astrocytes failed to cohabit with the axons; they physically damaged the axon layer by growing underneath them [150]. In the same study, however, reliable isolation of the six axonal compartments was demonstrated, making the device a proper tool for the study of localized biomolecular events in parallel.

Another group also designed a microfluidic platform for the segregated co-culturing of neuron cell bodies in one chamber and glial cells in another chamber, communicating together by very narrow channels through which axons are presumed to extend [151]. In addition to studying the localized interactions between axons specifically and the glial cells, this microdevice also aimed at providing the right setup for high resolution imaging of the interaction between a single axon with a single glial cell.

A microfluidic platform with two chambers allowing for the culture of two cell types was fitted with a pressure-controlled valve that either isolates the adjacent cell populations or allows for soluble mediator exchange, but also for direct cell-cell interactions [152]. Cells can either be grown in a 2D manner or embedded into biogels for 3D cultures. This platform allows for real-time, live-cell imaging of cells, and has been used for the study of synapse formation and also migration of tumor and endothelial cells during angiogenesis-intravasation/extravasation under hypoxic and normoxic conditions. With the similar goal of enabling optional communication between cells in different channels, Bianco and colleagues designed an overflow microfluidic network device to provide a realistic multi-cell system to mimic neuroinflammation and its role in neurodegenerative disease [153]. The authors wanted a system that is easy to use, that involves several cell types reflecting the complexity of brain cell interactions and that allows for proper analysis of cell morphology and electrophysiology. The overflow system gives the option of either keeping cells in different chambers completely isolated from each other or allowing for exchange of soluble factors and organelles between chambers. This device was used to examine the effect of astrocytes from distinct brain regions on cortical and hippocampal neuron viability, under inflammatory conditions. This system allowed the study of the interaction of multiple cell types and showed that astrocytes from different sites differentially modulate neurons challenged with inflammatory stimuli.

Finally, a microfluidic system was used to study Schwann cell-directed peripheral nerve regeneration following injury and transplantation [154]. The microplatform consisted of a neuron somatic body chamber connected to an axonal microchannel, which in turn communicates with another compartment where Schwann cells are grown. The neuron and Schwann cell compartments were of different capacities, thus allowing for a unidirectional flow and diffusion of solutes. Results from this study showed that even with the lack of axon and Schwann cell contact, Schwann cells can still direct axon growth. Data also suggested that adding glial cell line-derived neurotrophic factor (GDNF) could enhance peripheral nerve regeneration, even when transplanted Schwann cells and/or axonal parts come from mismatched sites.

### 2.4. Blood-Brain Barrier-on-a-Chip and Drug Delivery

**Figure 3:**
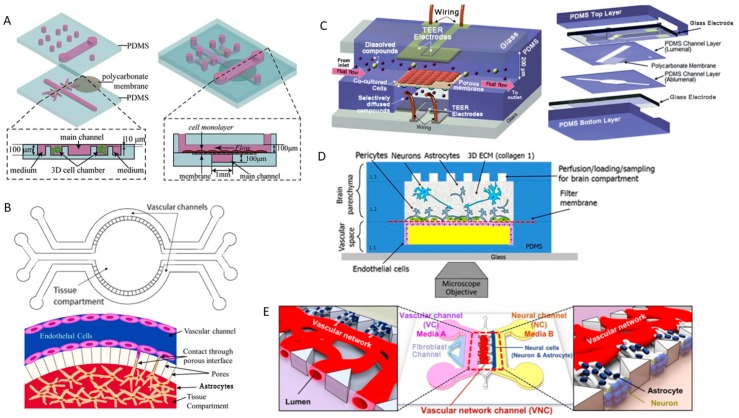
Schematic diagrams of blood-brain barrier (BBB)-on-chip models. (**A**) microchip BBB model [161]; (**B**) Neonatal BBB-on-chip [163]; (**C**) µBBB system [164]; (**D**) Neurovascular microfluidic bioreactor [165]; (**E**) 3D microfluidic neurovascular unit platform [166].

The BBB maintains and protects the CNS environment by regulating the exchange between the blood and the CNS. Endothelial cells found on the luminal side compose the barrier which is induced and maintained mainly by astrocytes [155]. Pericytes, microglia and neurons have also been shown to induce some BBB characteristics when cultured with endothelial cells [156]. The limited ability of drugs to cross the BBB presents a major difficulty in the treatment of CNS diseases [157]. Nanoscale particles can cross the BBB, mediating the development of therapeutic strategies [158]. In vitro BBB models have been used to investigate transport across the BBB and to study the mechanisms which regulate and maintain the BBB. A monoculture BBB microfluidic device was developed to study the permeability of the BBB under pulsed electric fields (PEFs) [159]. The device consists of six bottom parallel channels with a top channel running perpendicular to the bottom channels separated by a porous membrane. The top channel is lined with human cerebral microvascular endothelial cells (hCMECs) which comprise the BBB, and electrodes are placed at the openings of the bottom channel to produce the PEFs. The model showed that hCMECs prevented high molecular weight dextrans from crossing, but under PEFs, the tight junctions (TJs) were disrupted and allowed dextrans to cross. The study showed that regardless of the magnitude of the PEFs, increasing the pulse number beyond 10 decreased the viability of the hCMECs which led to irreversible effects. However, low amplitude PEFs with 10 pulses led to BBB permeability due to TJ deformation and allowed for complete recovery after treatment. Microfluidic models have gained much interest in this field due to the feasibility of applying physiological shear stress to endothelial cells which have shown to enhance barrier function [160]. A microfluidic platform was developed to mimic the flow of brain capillaries to enhance barrier function and simulate drug delivery to the brain and its efficacy. The microfluidic platform was comprised of two main channels placed on top of each other and separated by a porous membrane [161] (Figure 3A). The top channel represents the blood chamber and is placed perpendicular to the bottom channel which is the brain chamber. There are two other channels parallel to the bottom channel which are used to 3D culture the human glioblastoma cell line, U251, in agarose gels. The model permeability was validated by using sodium fluorescein and fluorescent dextran. The model was then tested for the permeability of sunitinib, which is known to cross the BBB and its toxicity to U251 cells was assessed. The model showed that sunitinib could cross the model BBB and that U251 cells cultured in 3D demonstrate a superior survival rate compared to 2D culture [161]. Yeon et al. developed a 2-channel microfluidic device utilizing human umbilical vein endothelial cells (HUVECs) [162]. The two channels were separated with a microhole array that was small and allowed a monolayer to be formed. The HUVECs were cultured with astrocyte-conditioned media (ACM) that induced TJ formation compared to cultures without ACM enhancing BBB-like properties. Permeability assay performed on the device showed that large molecular weight dextrans were not able to pass while smaller ones could with a decrease of permeability in HUVECs cultured with ACM. Deosarkar et al. developed a more biologically relevant BBB-on-chip which utilized astrocytes to further induce BBB characteristics. The model consisted of a central channel with porous interface which represents the brain compartment with two channels on either side representing the blood compartments [163] (Figure 3B). Rat brain capillary endothelial cells (RBECs) and rat neonatal astrocytes were cultured in the blood and brain compartments, respectively. The model showed significantly lower permeability of fluorescent dextran when compared to its Transwell counterpart. The model also showed that choosing the appropriate cell source plays a major role where neonatal RBECs improved model permeability and the transendothelial electrical resistance (TEER) compared to using adult RBECs [163]. Another BBB-on-chip was developed using an immortalized mouse brain capillary endothelial cell line, b.End3 in combination with a mouse astrocytic cell line, C8-D1A [164]. The device comprised two channels on top of each other separated by a porous membrane, and glass electrodes for real-time TEER measurements showed better TEER values and lower permeability coefficients (Figure 3C). The study showed that static co-culture of endothelial cells and astrocytes had better TEER than endothelial cells monocultured under dynamic conditions and that dynamic co-culture conditions showed the best results [164]. Brown et al. utilized microfluidic technology and 3D culture to create a BBB-on-a-chip that is more physiologically relevant. The device had a similar architecture to the previous model and used primary human brain microvascular endothelial cells in the luminal side and astrocytes and pericytes in the abluminal side with the addition of neurons derived from human induced pluripotent stem cells (hiPSCs), in a 3D collagen matrix [165] (Figure 3D). The model had high TEER measurements and low permeability to FITC dextran of different molecular weights. Tissue engineering strategies have also been utilized to create a membrane-free BBB-on-a-chip. The model consists of two channels and one middle chamber side by side [166] (Figure 3E). The middle chamber contained the endothelial cells while astrocytes and neurons are cultured in a fibrin hydrogel in the adjacent pockets. This allowed the astrocytes to be in direct contact with the endothelial cells while being spatially separated and was shown to further induce BBB like characteristics. The model was able to obtain close to in vivo results of permeability for 20 and 70 kDa FITC dextran [166]. Koo et al. developed a tetra-culture 3D BBB microfluidic device that was used to test the permeability and toxicity of organophosphate-based compounds [167]. The model was made on the 2-lane OrganoPlate using murine neuroblastoma cell line, N2a, C8-D1A and murine microglia cell line, BV-2 embedded in rat tail collagen type I gel with b.End3 cultured on the surface. The device utilizes capillary pressure barriers to separate the gel and the media. The model generated reproducible results which could be used to extrapolate in vivo results, reducing the need for animal models. BBB-on-a-chip models that closely resemble in vivo BBB models provide better insight into the factors involved in inducing and maintaining the BBB and allow an easy and reproducible means of studying pharmacological candidates to cross the BBB.

## 3. Nervous System Disease Models on Microplatforms to Replicate Cancer and Neurodegenerative Diseases

### 3.1. BBB Disruption and CNS Diseases

**Figure 4:**
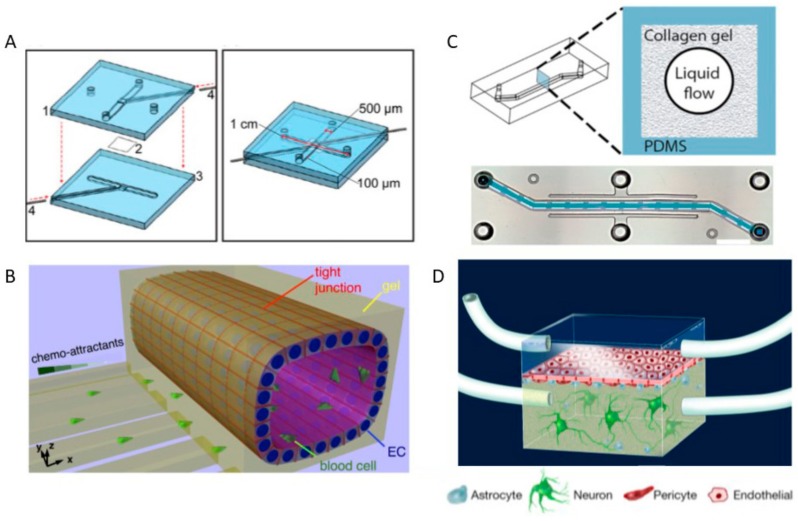
Schematic diagrams of BBB disruption models. (**A**) BBB chip. Figure adapted from [170]; (**B**) 3D in vitro BBB model [130]; (**C**) 3D microfluidic BBB chip [171]; (**D**) Neurovascular unit microfluidic device [172].

Disruption of the BBB by loss of TJs leads to impaired transport processes of molecules between the blood and brain [168]. Compromised BBB function has been implicated with various neuropathology, such as neuroinflammation and cerebral ischemia, and neurodegenerative diseases, such as Alzheimer’s disease (AD), Parkinson’s disease (PD) and multiple sclerosis [169]. The mechanisms of BBB breakdown and the resultant of its breakdown are not well understood. Models that can dissect the mechanisms of BBB disruption and study the effects of the disruption are essential in advancing research and effective therapeutic strategies. A small microfluidic BBB model was developed to study the effects of mechanical and biochemical stimulation of immortalized human brain endothelial cell line, hCMEC/D3 [170]. The microfluidic chip was comprised of two channels separated by a porous membrane with hCMEC/D3 cultured on the top channel. The model integrated electrodes in the top and bottom channels for TEER measurements (Figure 4A). The hCMEC/D3 was subjected to shear-stress and had TEER values three times greater than static conditions. The model was then used to study the effects of proinflammatory cytokine, tumor necrosis factor alpha (TNF-α). Stimulation by TNF-α decreased the TEER values by 10-fold, leading to the breakdown of the BBB. Another model was designed to study BBB disruption under neuroinflammation and ischemic conditions. The BBB model was a 3D microfluidic platform with a main vascular compartment and multiple smaller channels perpendicular to the main compartment [130]. The blood compartment was coated with collagen type I gel and cultured with rat brain endothelial cells, RBE4 (Figure 4B). The model was validated by showing that neutrophils could not cross the vascular compartment into the smaller channels by adding interleukin 8 (IL8) which primarily attracts neutrophils to sites of injury. The model probed the effect of TNF-α. Treatment of TNF-α led to the reduction and delocalization of TJ protein, zonula occludens-1 (ZO-1) on the cellular boundaries. The model was then used to study the effect of ischemia by oxygen-glucose deprivation. The effect of ischemia led to elevated reactive oxygen species (ROS) production and to significant loss of ZO-1 expression [130]. These two models contribute to the understanding of BBB functionality under health and disease. However, the models utilize monoculture and are lacking in terms of capturing the crucial role played by astrocytes and pericytes. A triculture model using human brain microvascular endothelial cells (hBMECs), astrocytes and pericytes was developed to investigate the underlying mechanisms of neuroinflammation (Figure 4C). The model was comprised of a single channel coated with collagen type I gel embedded with astrocytes and seeded with hBMECs on the surface of the gel or with pericytes and hBMECs seeded on the surface of the gel [171]. The model showed that co-culture with either astrocytes or pericytes increased barrier function by reducing the permeability of fluorescent dextran into the surrounding gel. The cells were treated with TNF-α and the cytokine release profiles were analyzed. The coculture condition when compared to hBMECs alone had higher release of IL6, IL8 and granulocyte colony stimulating factor (G-CSF). IL6 and G-CSF are responsible for neuroactivation and neuroprotection, signifying the contribution of astrocytes and pericytes in understanding BBB disruption and maintenance. A brain-on-chip was designed to study the effects of neuroinflammation to the BBB as well as neurons. The chip was designed with two channels separated with a porous membrane culturing hBMECs, astrocytes, pericytes and neurons [172]. The hBMECs were cultured on the top of the porous membrane and astrocytes and pericytes on the bottom (Figure 4D). Neurons derived from pluripotent stem cells were later introduced in a collagen gel below the astrocytes and pericytes. The chip modeled inflammation by adding lipopolysaccharide (LPS), which represents a systemic infection, or adding a cytokine cocktail. The addition of LPS or the cytokine cocktail both resulted in BBB breakdown, but the BBB was able to regain its function under the LPS condition. The brain-on-chip was able to observe that cytokines in the top (vascular) compartment and bottom compartments were differentially elevated at later stages of LPS or cytokine cocktail exposure. These models are crucial in mapping metabolic pathways and responses of different environments which will lead to new insights in developing therapeutic strategies.

### 3.2. Brain Cancer and Metastasis

Brain cancer is among the leading causes of cancer death [173]. The most common and fatal type of brain cancers are gliomas [174]. Limitations of 2D models in studying brain cancer and metastasis have prompted the development of in vitro models that better mimic the physiological microenvironment of the nervous tissue. Co-culture in Transwell-based models is a better tool to study cellular interactions and movements than traditional 2D cultures despite several limitations such as the lack of flow and potential gaps between endothelial cells, affecting cell morphology and barrier efficiency [163,175,176,177,178,179], but also the fact that a stagnant water-based medium enhances the permeability of water-soluble molecules rather than lipid-soluble molecules [180,181]. Neurons and their microenvironment play a critical role in brain cancer, metastasis and disease progression [182,183]. Brain-on-chip and microfluidic models have emerged, in an attempt to close the gap between in vitro and in vivo models.

A 3D microfluidic system with two compartments was developed by Ma et al. to test the cytotoxic effect of the anti-cancer drug (temozolomide) and prodrug (ifosfamide) on glioblastoma multiforme (GBM) cell line, M059K and their metabolism in HepG2 liver cell line and its derivative C3A [184]. Liver and GBM cells were grown in 2D setting and in 3D polylactic acid (PLA) scaffolds in either compartment of the microfluidic system. Liver cells reduced the activity of temozolomide and activated the ifosfamide prodrug. M059K cells grown in the 3D system showed higher resistance to both drugs, when compared to their 2D counterparts. This observation emphasizes the fact that cells lines that would respond to treatments in ‘flat’ cultures might show greater resistance in 3D cultures; hence the importance of designing proper models for drug testing that more closely resemble in vivo settings. Lee and colleagues developed a microfluidic chip to investigate glioma cell alignment and migration at the interface of microvessels [185]. The microfluidic chip with one compartment integrated hyaluronic acid (HA) hydrogel embedded with the glioma cell line, A172 and was separated from the channel by a porous membrane. The HA hydrogel was cross-linked using matrix metalloproteinase-sensitive peptides to study the cells’ ability to degrade the hydrogel and also arginyl-glycyl-aspartic acid (RGD) peptides to assess cellular attachment. The model showed that under static conditions, cells remained mostly rounded and induced no scaffold remodeling, while under flow, cells became elongated and aligned with the direction of the flow. Cells on the upper part of the hydrogel migrated towards lower levels with higher concentration of nutrients. Furthermore, the addition of vascular endothelial growth factor (VEGF) to the medium induced a fivefold cell size increase and extensive spreading, compared to cells in VEGF-free medium. Cells under static conditions showed no change in morphology upon exposure to VEGF, highlighting the importance of the combination of dynamic conditions and growth factors. In 2014, Tourovskaia et al. designed a microchip to model angiogenesis and the BBB [186]. The angiogenesis model consisted of two tubular channels separated by a collagen matrix with or without pericytes. One channel was populated with human endothelial cells (parent channel) and the other channel with pro-angiogenic (sprouting) factors: VEGF, basic-fibroblast growth factor (b-FGF) and phorbol-12-myristate-13-acetate (PMA). Within 24 h, cells from the parent channels had started ‘sprouting’ and migrating towards the channel with growth factors, spanning the collagen matrix. Moreover, initially homogenously distributed pericytes were found to become associated with sprouting vessels, in addition to depositing basement membrane together with the endothelial cells. To reproduce the BBB, this microfluidic system was slightly modified to consist of only one channel embedded in a collagen matrix populated with human astrocytes and pericytes. The channel was populated with brain microvascular cells or dermal microvascular cells. Once cells had adhered, the channel was continuously perfused. Results showed complete coverage of the inner channel wall with microvascular cells, which also formed the correct junctions. Matrix astrocytes and pericytes also organized themselves in a way resembling the in vivo situation. Bovine serum albumin (BSA) was used to test for the paracellular permeability of the created microvessels, compared to control channels not populated with endothelial cells. Close to 75% of tested microvessels were impermeable to BSA, matching reports from the literature [187,188]. The group then combined both models to study cancer cell migration and extravasation; they generated sprouting vessels, populated with fluorescent cancer cells. Highly metastatic PC3 prostate cancer cells, but not BT-474 breast cancer cells with low metastatic potential, were found to have left the microvessel. Therefore, the authors concluded the relevance of their microfluidic system in mimicking angiogenesis and barrier function, as well as serving as a good model to study cancer metastasis to the brain. In 2015, a group used the microfluidic device μLane to image the real-time migratory responses of individual medulloblastoma cells within microenvironments of defined epidermal growth factor (EGF) and stromal cell-derived factor 1-alpha (SDF-1) gradient profiles. Both EGF and SDF-1 are major chemoattractants of tumor cells in the CNS [189]. μLane applies convective-diffusion mechanics to maintain controlled gradients of given solutes. Briefly, it consists of a closed microchannel connecting two 9 μL-fluidic reservoirs, on top of which another system of two 170 μL-chambers connected by an open bridge maintains the hydrostatic balance. Both chambers (of the upper system) communicate with the two reservoirs (of the lower system). Results showed that medulloblastoma cells gain motility at initially elevated concentrations of EGF (mimicking the paracrine situation) and become less motile with decreasing concentration gradient fields. The group explained that their system induces medulloblastoma cells into becoming motile at high EGF concentrations, but that this motility is slowed down as migrating cells approach fields with lower gradients (despite possible greater chemoattractant concentrations). Lei and colleagues created a compartmentalized microfluidic device (CMD) to study the interaction between cortical neurons and different cancer cell lines [190]. The neurons and cancer cells were cultured in the different compartments, separated by connecting channels traversed by neurites. The model showed that the presence of neurites enhanced cancer migration towards the neural compartment but disrupted neurites, inhibited neural signaling, and hindered cancer migration. This study highlights the role of neurites (and neuronal communication) in modulating cancer progression and metastasis, and their relevance to microfluidic systems mimicking the brain microenvironment. Another group designed a microfluidic device to mimic the BBB, attempting to reproduce its physiology in all its complexity [191]. They put together 16 parallel systems operating simultaneously. Each functional unit consisted of a micro-channel with four uniform BBB regions. Each region consisted of a vascular compartment and a brain compartment (extracellular matrix (ECM) collagen, primary astrocytes and endothelial cells). Barrier strength was assessed by the expression of TJ proteins in the endothelial cells, all of which were upregulated in the presence of astrocytes and under dynamic flow. Moreover, the TEER reached an exceptionally high value far exceeding the TEER reported for Transwell-based BBB models. A permeable hydrophilic sodium fluorescein-labeled dye was used to evaluate the efficiency of the barrier of this BBB system, both under flow and static conditions. A complete BBB (endothelial cells, astrocytes and matrix) completely blocked dye transfer from the vascular compartment to the brain compartment, under flow or static conditions, while endothelial cells alone allowed dye transfer, illustrating the importance of an intact BBB in preventing foreign substances (and tumor cells) from reaching the brain matter. Lung adenocarcinoma (A549), breast cancer (MDA-MB-231), melanoma (M624) and liver (BEL-7402) were introduced into the vascular compartment. All three former cancer cell types disrupted the BBB, while liver cancer cells did not. Lung and breast cancer cells showed greater migration than melanoma cells, while liver cancer cells did not seem to cross the BBB over 72 h. The brain compartment was then populated with U87 glioma cells, which could not cross the BBB from the brain into the vascular compartment under flow conditions. The administration of drugs to induce apoptosis of U87 into the vascular compartment showed the exclusive effect of a lipophilic drug, while water-soluble compounds could not reach the brain compartment. This work concluded the importance of astrocytes and endothelial cells together in providing BBB integrity and preventing metastasis of the brain and the involvement of astrocytes in preventing brain metastases. In fact, other studies have also suggested that astrocyte-conditioned ECM applied to endothelial cells was enough to induce the expression of TJ proteins, sealing together the endothelial cells and creating higher TEER values [169,171,178,192]. One limitation of this microfluidic system is the use of rat cells, which only modestly reproduce the human situation.

A microplatform was designed by the same group in 2016 to study the behavior of lung adenocarcinoma A549 cells in the presence of other organ-specific cells. Bronchial epithelial, lung cancer, endothelial, mononuclear, and fibroblast cells were grown separated by the biomembrane, while astrocytes, osteocytes, and hepatocytes were grown in distant chambers, to mimic lung cancer cell metastasis to the brain, bone, and liver. This multiorgan-device reproduced in vitro scenarios where lung cancer cells metastasize to the brain, bone and liver and change their molecular profile [193]. The same group evaluated the adhesion/extravasation of the lung adenocarcinoma cell line (A549) across a TNF-alpha induced inflammatory microvasculature, in a micro-device. Results showed that A549 cells increased adhesion to the inflammatory endothelium [194]. Terrell-Hall et al. characterized another less complex microfluidic system [195] where human vein endothelial cells were cultured with CTX-TNA2 rat astrocytes for the establishment of a BBB or with Met-1 metastatic murine breast cancer cells to establish a blood-tumor barrier (BTB). The major aim was to assess the permeability of both systems to potential anticancer drugs, given that tumor cells somewhat disrupt the BBB, but the BTB still retains its selective impermeability to drugs, as well as a potent efflux by P-glycoprotein. The authors designed a microfluidic device with peripheral endothelial cells and either astrocytes or tumor cells in a central compartment. Dynamic flow was applied to the outer peripheral compartment to mimic physiological fluid flow conditions. Confocal microscopy 3D reconstruction demonstrated 360° coating of outer tube-like compartment with endothelial cells. Light microscopy showed morphological changes of endothelial and central compartment cells in the presence and absence of dynamic flow, further demonstrating the relatively more modest relevance of Transwell systems in the study of blood-brain exchanges. This same model was used test the permeability of the BBB and BTB to trastuzumab antibody, showing that both the BBB and the BTB were permeable to the antibody, though in small quantities, likely below effective levels [196].

Shumakovich et al. described the behavior of breast cancer cell lines in ACM, in order to study the mechanisms underlying preferential metastasis of breast tumors to the brain [197]. They confirmed previous works reporting the chemoattractant role of astrocytes (and ACM) in directing tumor cell migration from breast primary tumors through the blood stream and extravasation in the brain. They described the involvement of matrix metalloproteinases contained in the ACM, the altered morphology and increased 2D migration speed of breast cancer cells towards ACM-rich media, the modulation of migration speed on substrates containing different extracellular proteins (fibronectin, laminin, different collagens, etc.). The group then challenged the common mistake of tending to extrapolate findings of in vitro isolated assays to the real situation occurring in vivo. More specifically, the authors wondered whether the chemoattractant effect of ACM described in flat 2D cultures would similarly hold for breast cancer cells having to travel along increasingly narrower capillaries in the brain tissue. They used a four-inlet microfluidic device with different channel widths of 3, 10 and 50 µm to evaluate the migratory ability of breast cancer cells in response to serum-free or serum-containing ACM; serum being a natural attractant to breast cancer cells. In designing their ‘confined’ microfluidic model, the authors focused on two aspects of tumor cell migration in the blood stream: guidance provided by tumor cells touching walls of the capillaries and the extent of nuclear deformity required for tumor cells to squeeze themselves in narrow pipes. Briefly, they populated the lowermost channel with breast cancer cells and the uppermost channel with either ACM (to assess the chemoattractant effect of ACM in a confinement) or with serum-containing media or serum-containing ACM (to check if ACM would enhance the chemoattractant effect of the serum). The 10-µm wide channels provided the best balance between lateral guidance and nuclear deformity, with the highest movement speed of cells, compared to narrower and wider channels. Interestingly, the chemoattractant effect of the ACM dramatically shown in 2D assays was contrasted in microfluidic confinement, where the migratory speed of cells was comparable towards ACM or regular serum-containing media. This model could serve for studying the preferential attraction of metastatic cells towards specific environments, and how strongly tissue-specific extracellular deposits determine the ability of tumor cells to travel through tiny spaces and reach destination.

### 3.3. Alzheimer’s and Parkinson’s Diseases

Alzheimer’s disease is an irreversible, progressive brain disorder that slowly destroys memory and thinking skills and, eventually, the ability to carry out the simplest tasks. Amyloid-β (Aβ) peptides, intracellular accumulation and hyperphosphorylation of tau protein, as well as a variety of other protein fragments, have been demonstrated to play a key role in the pathological changes underlying AD [198,199,200,201,202]. Parkinson’s disease is another chronic progressive neurodegenerative disorder of movement that is accompanied by other non-motor features as cognitive impairment, autonomic dysfunction, disorders of sleep, depression and hyposmia (impaired smell) [203,204]. The pathological definition of PD is loss or degeneration of the dopaminergic neurons in the substantia nigra and development of Lewy Bodies (a pathologic hallmark) in dopaminergic neurons [203,205,206]. Lewy Bodies, or abnormal intracellular aggregates, contain several proteins including alpha-synuclein (α–Syn) and ubiquitin that leads to the impairment of neuronal functioning [203].

The degeneration of neurons in AD and PD leads to the dysfunction and the disruption of the neuronal networks in which neurons are embedded [207]. This is triggered not only by cell death but also by changes in the neuronal microenvironment and the interactions between the various cell types that are embedded in the network: neurons, astrocytes, endothelial cells, pericytes, etc. Microfabrication techniques that are based on designing elastomeric polymers have enabled the development of microfluidic devices that helped direct tackling of neurodegenerative diseases [208]. In particular, through the diffusion of soluble factors [209] and the exposure to cues that have temporal and spatial variability, intricate studies of the interactions between different neurons as well as interactions between specific neuronal parts (axon-glia [150], axon-neurons [210]) are doable in a controllable and reproducible fashion [33]. This is important in studying these disease as biomimetic microsystems allow the reproducibility of in vitro neuron-to-neuron communication, which is the hallmark of AD and PD. Models based on 2D and 3D cultures of neuronal cells were thus developed to test neuro-cytotoxicity and degeneration [39,207,211] where dissociated mesencephalic neurons have been extracted from fetal rats [212], DA-neuron-derived cell lines [213], and embryonic stem cell-derived dopaminergic neurons [214]. Biological processes such as cell differentiation [150,215], neurite extension of neuronal cells [216,217], and cell migration (e.g., neutrophil chemotaxis [218] and cancer cell migration [60]), which are affected by chemical concentration gradients, have been widely studied using the microfluidic devices. CMDs also enable superior spatiotemporal control and have been widely used to study neuron–glia interactions [217,219].

AD models can be generated by culturing neurons that are differentiated from hiPS cells derived from fibroblasts of AD patients. Using neuronal cells expressing Aβ differentiated from the NSCs of the patient, the molecular mechanisms of AD can be induced [216]. This has significant implications on the neuroscience community as it enables the application of several electrophysiological and histological techniques to the cultured neurons and their microenvironment to study the intrinsic and extrinsic labyrinths of the disease. Choi et al. developed a microfluidic device with a very slow flow rate to mimic interstitial flow in brain tissue to study the toxicity of Aβ oligomeric assemblies [220]. Osmotic pressure was the driving force of the device by placing a water-filled PDMS cube with a cellulose porous membrane in a Petri dish with polyethylene glycol solution. Neuronal cells were cultured in the microfluidic channel and were exposed to oligomeric assemblies of Aβ and fibrillogenesis was observed. The study showed that fibrils of Aβ did not significantly influence cell viability but the exposure to oligomeric assemblies produced the neurotoxicity. Two-chamber CMDs which can separate the soma and axon, have been used to study axonal transport in the presence of Aβ [221]. The study showed that Aβ significantly reduced the acetylation of α-tubulin acetylation which is responsible for mitochondrial transport as well as decreasing the length of mitochondria. Tubastatin A (TBA) was used as the HDAC6 inhibitor to increase α-tubulin acetylation and was shown to reverse the effects of Aβ on mitochondria length and motility. Another study also used a two-chamber CMD to study neurotoxicity and axonal degeneration in the presence of Aβ and glutamate [222]. The study showed that the combination of memantine with vitamin D was able to prevent axonal degeneration and can be used as a potential treatment to delay AD progression. Moreover, the microscale dimensionality and flexibility of microfluidic systems enable the capturing of the detailed behavior of the dynamics of such diseases. Ruiz et al. developed a four-chamber CMD to study the effects of Aβ on neurons and the contribution of microglia [208]. Primary rat neurons and microglia were extracted and plated in the four chambers with different conditions. The study showed that the presence of microglia reduced the damage of neurons exposed to Aβ. They also enabled assessment of the fine spatiotemporal details of biochemical and mechanical stimuli over elements of the cellular environment. Of particular importance, the CMD enabled the easy application of electrical and optical stimulation, for example, by implanting electrodes on the bottom of the chamber to measure neuronal signals in AD or PD cultured slices. Microfluidic chips operated by gravity-induced flow have been also designed to study AD [208]. Stimuli (Aβ species) are applied and distributed to chambers via perfusion, allowing direct demonstration of the high toxicity of oligomeric Aβ compared to the fibrillar form. MMSAs (microfluidics-based mobility shift assay) were developed and applied for the screening of β-secretase (BACE1) inhibitors for AD, which initiates the generation of toxic Aβ from amyloid-β precursor protein (APP) [223]. In this recent study, BACE1 activity assay was established with a new fluorescent peptide substrate, and high-quality ratiometric data were generated in both endpoint and kinetic modes that enabled further studies on the mechanism of inhibitors under kinetic mode [223].

PD can be induced via a culture model of primary neurons in the presence of fibrils of α-Syn in microfluidic devices with microgrooves [207]. In these experiments, neurons are able to internalize and transport α-Syn fibrils along their axons to the soma; both mechanisms are similar to the characteristic patterns of Lewy bodies spread in vivo. CMDs have been used to integrate α-Syn transport tracking that would allow better analysis of the neural activity and the underlying neural circuitry to investigate the pathogenesis of PD [224,225]. To make these models more realistic, one can further integrate microfluidic features such as microvalves to control fluid routing and other signaling pathways between the various neurons in the culture.

### 3.4. Axon Regeneration and Neural Cell Biology

**Figure 5:**
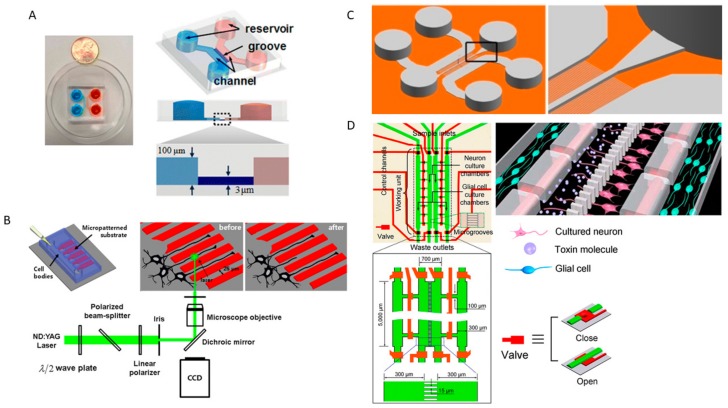
Schematic diagrams of axonal injury and regeneration models. (**A**) Two-chamber CMD. Figure adapted from [230]; (**B**) Pulsed laser microbeam integrated microfluidic device. Figure adapted from [230]; (**C**) Three-chamber CMD [232]; (**D**) Multi-chamber CMD controlled with valves. Figure adapted from [233].

Peripheral nerves that have suffered axonal injury can regenerate the injured axon and reinnervate their target even in cases of axotomy [226]. However, axonal regeneration is not found in the CNS when axonal injuries occur [227]. Neuronal survival and axon regeneration processes involve a myriad of intrinsic and extrinsic factors [228]. The superior regenerative properties of the PNS are caused by their more suitable microenvironment which permits axonal regeneration following injury unlike the CNS [229]. Compartmentalized lab-on-chip systems have become a very valuable tool to culture CNS neurons and to study neurodegeneration and neuroregeneration. Different lab-on-chip systems have been engineered to study CNS axotomy by applying physical forces or chemicals agents [230]. A two-chamber CMD was developed to study CNS axotomy by vacuum aspiration of the axonal chamber [217]. The device had two chambers separated by microgrooves running perpendicular to the main chambers (Figure 5A). The device isolates axons by directing axonal growth from one compartment to the other by using fluidic flow through the microgrooves. Axotomy is achieved by vacuum aspiration of the axonal compartment. The device provides insight into axonal injury and regeneration. An integrated microfluidic devices utilizing lasers to precisely induce axotomy of single axons was developed using a pulsed laser microbeam [231]. The device reproduces Wallerian degeneration where the severed axon degenerates. The device contained a neuron culture compartment with channels to guide axonal growth (Figure 5B). Complete and partial axotomy of embryonic rat cortex neurons was carried out in normal culture medium and in ethylene glycol-bis(P-aminoethyl ether)-*N*,*N*-tetraacetic acid (EGTA, a calcium-specific chelant) treated media. The study showed that axonal degeneration in partial axotomy was reduced and regeneration was observed, especially in EGTA-treated media [231]. This further validates the role of calcium ions influx in neuronal cell death. Another lab-on-chip device was developed to model neurons in the CNS undergoing chemically induced axotomy. The device was composed of three compartments separated with microchannels [232]. Neurons derived from embryonic mice were seeded in one compartment while they developed axons that crossed through the other two compartments (Figure 5C). A detergent was then added to the central compartment to sever the axons. The model mimicked features of the Wallerian degeneration such as distal axon fragmentation. The model also showed that distal axon degeneration was delayed by β-Nicotinamide adenine dinucleotide hydrate (βNAD) [232]. Another compartmentalized model was developed to investigate the role of glial cells in neuronal injury repair. The model used neurons, astrocytes and Schwann cells taken from Sprague-Dawley rat pups and was composed of four compartments: (i) glial cell culture, (ii) neuron culture, (iii) axonal isolation, and (iv) glial cell culture which was separated by microchannels (Figure 5D) [233]. Axonal injury was achieved by applying a chemical, acrylamide (ACR) into either the axonal isolation or neuron culture compartments. When the neurons were co-cultured with glial cells, their survival rate increased and axonal regeneration was observed, indicating the secretion of trophic factors from cells since they were not in contact with the neurons. Axonal regeneration was achieved due to the microtubules remaining intact after ACR treatment. This provides insight into understanding the transport machinery responsible for transporting material blocks for regeneration.

## 4. Conclusions and Future Directions

Microplatforms represent an excellent device to create biomimetic models for nervous tissues and to perform basic and applied investigations on neural health and disease. Organ-on-a-chip systems enable the control of parameters including media flow, nutrients, test agents, cell-cell and cell-matrix interactions. These platforms also permit the analysis of cellular responses to mechanical, chemical and electrical stimuli while assessing cellular responses in situ. Organ-on-a-chip systems represent a close approximation of the in vivo environment and thus provide more realistic cell responses when compared to the commonly adopted 2D cultures. The ability to precisely control flows and stimuli in an automated manner improves standardization and reproducibility, leading to more precise outcomes in systematic studies. Organ-on-a-chip tools have been successfully used to enhance our understanding of the physiology of the NMJ, neurogenesis, synaptic formation and neuro-glial interactions. The microplatforms have also been used to mimic the BBB and investigate its role in drug delivery and CNS disease. In addition, organ-on-a-chip systems have been used to study brain cancer, metastasis, degenerative diseases, and axonal regeneration.

Despite being more biomimetic than traditional 2D culture systems, organ-on-a-chip systems still require significant improvement to replace in vivo studies. Although it is already common knowledge that 3D cultures are more representative than 2D, many organ-on-a-chip systems are based on 2D growth of cells. Achieving more representative models requires the use of 3D culturing tools, which have witnessed in the past few decades a major leap in both methods and materials. The literature is rich with biocompatible synthetic and natural biomaterials that can be prepared with a variety of methods, with high precision (e.g., bioprinting and two-photon lithography among others). The chemistry used in biomaterials preparation can also be mild to elicit minimal or no cell response so that cells are encapsulated in biomaterials and cultured for long periods. Another area of focus is in situ stimulation and activity recording, which is of particular importance in nervous tissues. Most organ-on-a-chip systems currently focus on investigating specific cell behavior without taking in consideration the importance of neural activity, which is integral for neuronal response. The systems also do not record neural activity except for very few cases. Lab-on-chip (LOC) and organ-on-a-chip systems may be designed to enable recording and stimulation of neurons in precise predefined locations to gain more insight into neural responses. This can be achieved by using conductive materials in the preparation of the platforms and micropatterning cells and corresponding stimulation/recoding spots. Finally, although one of the main goals of organ-on-a-chip systems is to mimic the in vivo scenario and ultimately replace/reduce animal models, rarely do organ-on-a-chip studies provide a systematic comparative analysis of the developed system with an in vivo model. A systematic comparison between developed organ-on-a-chip systems and an in vivo model would provide a very strong statement to the relevance of the organ-on-a-chip model.

The integration of patient-derived cells with microfluidic devices to create realistic neurodegenerative disease microenvironments has huge scientific implications. This could have an even stronger impact when ECM biomaterials are used along with the induction of biochemical and mechanical factors to enable various electrophysiological and histological approaches. Microfluidic devices exhibit the capability of integrating these components into small LOC style devices that could meet a variety of needs from basic science to translational drug discovery.

## Figures and Tables

**Table 1:** Differences between two-dimensional (2D) and three-dimensional (3D) culture systems [39,40,41,42].

2D Cell Culture	Cellular Characteristics	3D Cell Culture
Flat and stretched cells on monolayer	Morphology	Form natural shape in aggregate or spheroid structures
Faster rate than in vivo	Proliferation	Depends on the cell type and 3D model system
Exhibits differential gene/protein expression levels	Gene/Protein Expression	Similar to in vivo tissue models
Only on edges	Cell-to-Cell contact	Dominant
Most cells are at the same stage (usually proliferating stage)	Stage of Cell Cycle	Different stages: proliferating, hypoxia, and necrotic cells
Grow and adhere on a flat substrate	Growth Conditions	Grow on matrix or in suspension media
No	Diffusion gradient of O_2_, nutrients, drugs, waste	Yes
No	Show resistivity to anticancer drugs	Yes
No	Mimicking in vivo environment	Yes
